# Supplementary material for: Lack of Phylogenetic Differences in Ectomycorrhizal Fungi among Distinct Mediterranean Pine Forest Habitats
Source: J Fungi (Basel). 2021 Sep 24;7(10):793. doi: 10.3390/jof7100793 (PMC8538088; doi:10.3390/jof7100793)
Supplement: Supplementary file 1 [file jof-07-00793-s001.zip › jof-1308271-supplementary.pdf]

Supplementary tables and figures

Table S1. Table summarizing the main texture and moisture properties of the study plots: Fine clay content (%), Clay content (%), Sand content (%), Moisture (g water/g soil). Ps: *P. sylvestris*, Pn: *P. nigra*, Ph: *P. halepensis*, Ps-Pn: *P.sylvestris-nigra*, Pn-Ph: *P. nigra-halepensis.*

Forest type	Range	Fine Lime^{-1}	Clay	Sand	Moisture
Ps	Min.	6.8	5	24.8	0.54
(14)	Mean	25.4	15.7	47.2	2.22
	Max.	44.9	31.5	83.4	4.5
Pn	Min.	21.5	3.30	24.7	1.19
(14)	Mean	27.95	14.92	44.04	2.13
	Max.	38.5	37.30	54.90	4.24
Ph	Min.	17.30	6.5	51.10	1.2
(4)	Mean	19.75	8.95	57	1.61
	Max.	23.20	11.40	64.80	2.70
Ps–Pn	Min.	7.50	9	25.10	0.78
(7)	Mean	21.10	18	49	1.80
	Max.	31.90	29.5	78.90	2.70
Pn–Ph	Min.	32.90	20.10	16.20	1.30
(3)	Mean	138.23	23.63	24.50	1.36
	Max.	43.70	29.40	30.90	1.44

Table S2. Most abundant ectomycorrhizal species detected in pure and mixed stands of *Pinus spp*.

P. halepensis	***P. nigra–P. halepensis***	***P. nigra***	***P. sylvestris–P. nigra***	***P. sylvestris***
Suillus spp.	*Suillus* spp.	*Suillus* spp.	*Suillus* spp.	*Hydnum repandum*
Phellodon niger	*Phellodon niger*	*Phellodon niger*	*Phellodon niger*	*Phellodon niger*
Rhizopogon mohelnensis	*Inocybe* spp.	*Tricholoma* spp.	*Tricholoma* spp.	*Inocybe ochroalba*
Hydnum spp.	*Lactarius sanguifluus*	*Hydnum repandum*	*Hydnum repandum*	*Tricholoma terreum*
Russula pallidospora	*Cortinarius hydrobivelus*	*Russula* spp.	*Russula pallidospora*	*Russula caerulea*
Inocybe ochroalba	*Russula pesudoaeruginea*	*Inocybe* spp.	*Inocybe ochroalba*	*Suillus* spp.
Cortinarius vernus	*Craterellus lutescens*	*Craterellus cornucopioides*	*Rhizopogon molhensis*	*Rhizopogon molhensis*
Tomentella subclavigera	*Boletopsis* sp.	*Boletus edulis*	*Inocybe asterospora*	*Russula* spp.
Lactarius sanguifluus	*Lactarius deliciosus*	*Lactarius sanguifluus*	*Sebacina flagelliformis*	*Lactarius sanguifluus*
Craterellus lutescens	*Tricholoma* sp.	*Tomentella* spp.	*Tricholoma terreum*	*Craterellus lutescens*
Russula delica	*Rhizopogon mohelnensis*	*Lactarius deliciosus*	*Craterellus lutescens*	*Lactarius deliciosus*

Hydnum repandum	*Boletus edulis*	*Hydnellum* spp.	*Boletopsis* spp.	*Sebacina* spp.
Lactarius deliciosus	*Cortinarius vernus*	*Sebacina* spp.	*Sebacina cystidiata*	*Boletus edulis*

Table S3. Phylogenetic species turnover, nestedness and total beta diversity values across host tree species stands.

Tree host	***Phylo β sim***	***Phylo β sne***	***Phylo β sor***
P.halepensis	0.54	0.09	0.63
P.nigra	0.79	0.06	0.85
P.sylvestris	0.79	0.06	0.85
P.nigra-halepensis	0.40	0.14	0.54
P.sylvestris-nigra	0.71	0.06	0.78

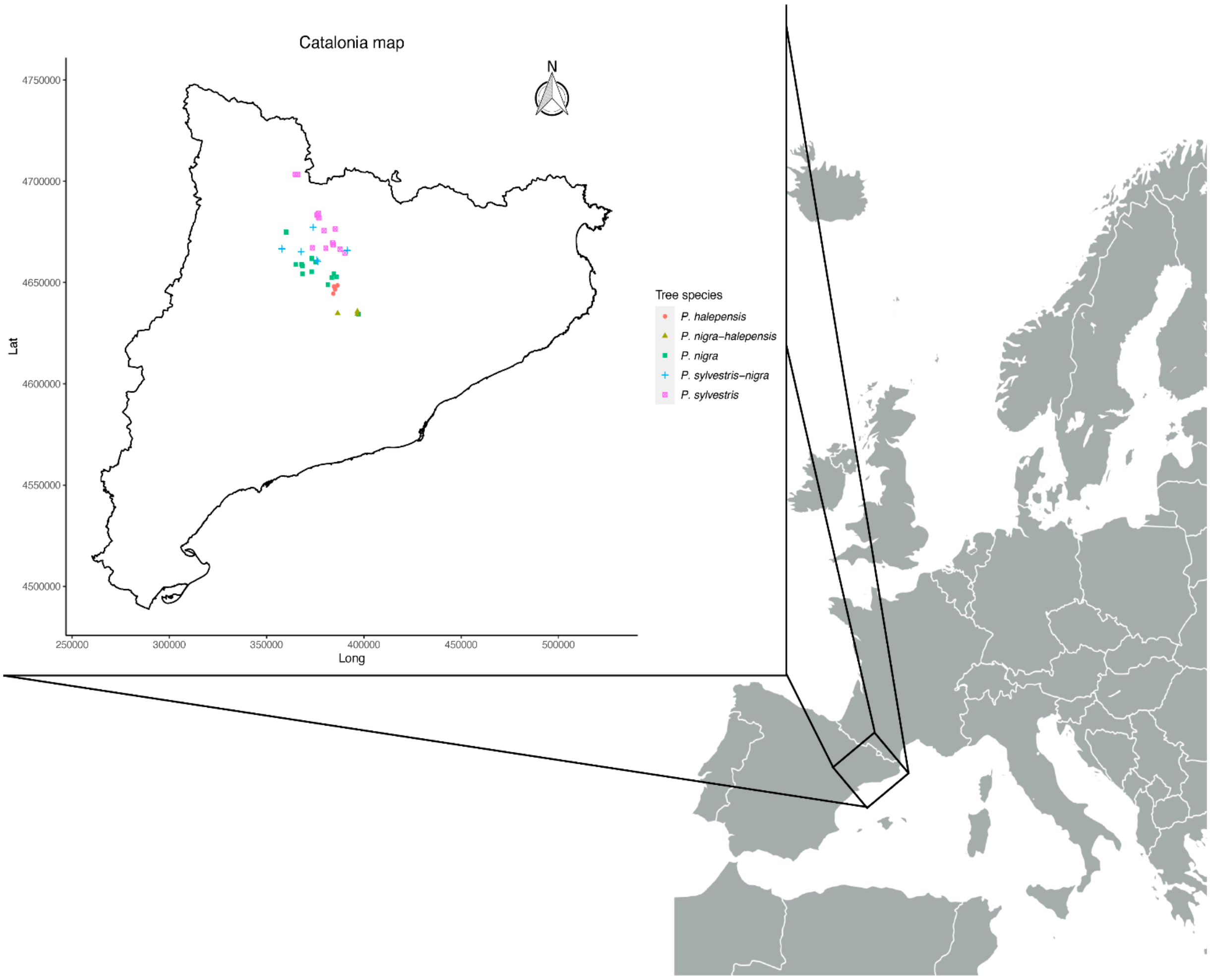

Fig. S1. Catalonia map displaying the location of the 42 plots. Colours and shapes correspond to pine tree species: *P. halepensis*, *P. nigra-halepensis*, *P. nigra*, *P. sylvestris-nigra*, *P. sylvestris*.

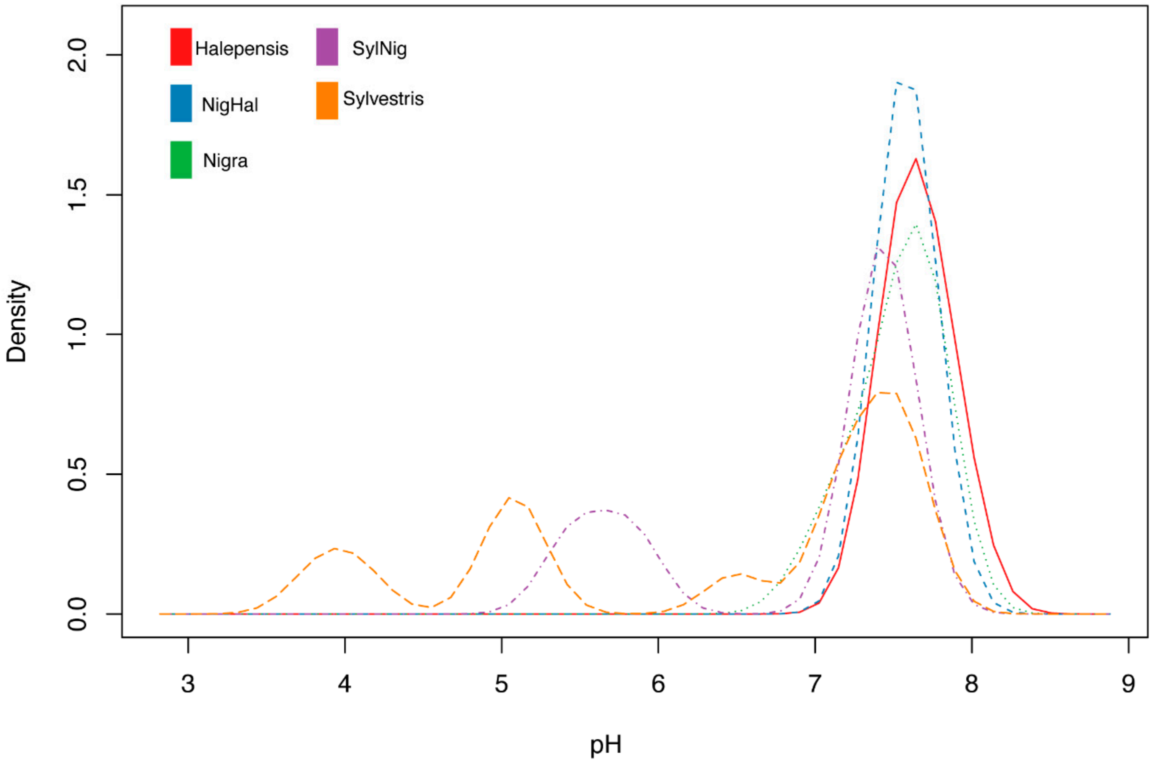

Fig. S2. Density curves of pH values of the five tree hosts from the permutational test of density equality (p-value: 0.04)

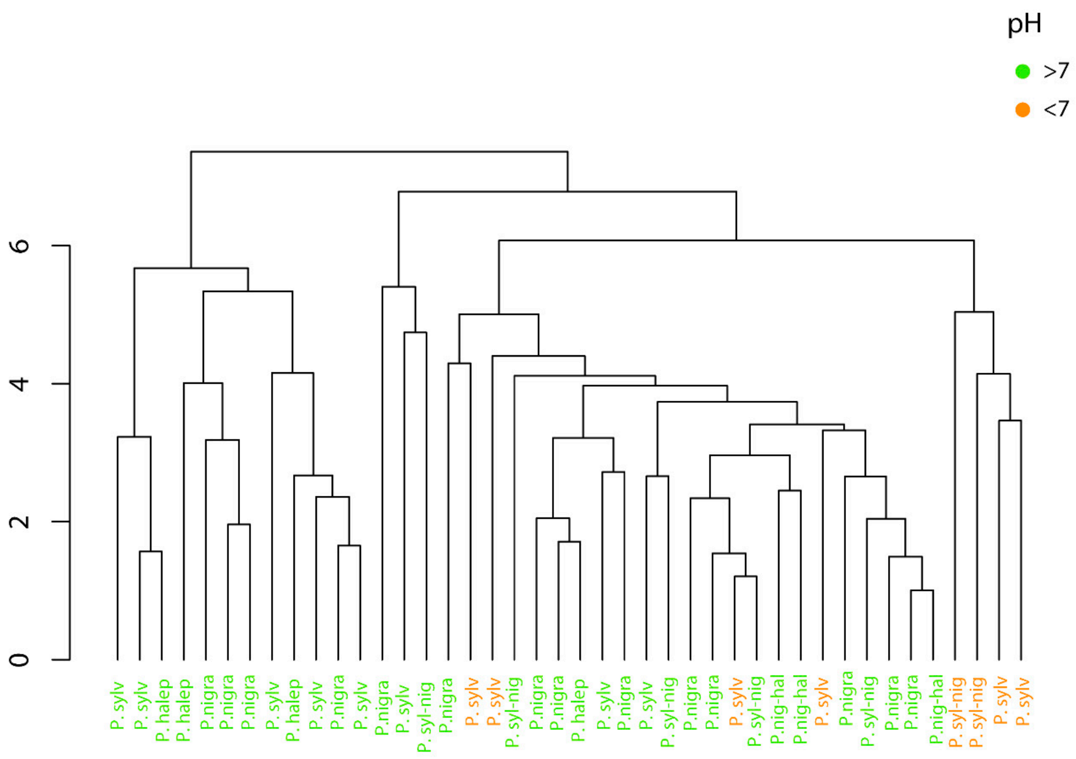

Fig. S3. Hierarchical clustering of ectomycorrhizal phylogenetic compositional data based on a Euclidean distance matrix. Tree host species are colored by pH values >7 (green) and pH values <7 (orange).
